# End-tidal capnography monitoring in infants ventilated on the neonatal intensive care unit

**DOI:** 10.1038/s41372-021-00978-y

**Published:** 2021-03-01

**Authors:** Emma Williams, Theodore Dassios, Niamh O’Reilly, Alison Walsh, Anne Greenough

**Affiliations:** 1 Department of Women and Children’s Health, School of Life Course Sciences, Faculty of Life Sciences and Medicine, King’s College London, London, UK; 2 The Asthma UK Centre for Allergic Mechanisms in Asthma, King’s College London, London, UK; 3 Neonatal Intensive Care Unit, King’s College Hospital NHS Foundation Trust, London, UK; 4 NIHR Biomedical Research Centre based at Guy’s and St Thomas’ NHS Foundation Trust and King’s College London, London, UK

**Keywords:** Risk factors, Outcomes research

## Abstract

**Objective:**

To assess whether end-tidal capnography (EtCO_2_) monitoring reduced the magnitude of difference in carbon dioxide (CO_2_) levels and the number of blood gases in ventilated infants.

**Study design:**

A case–control study of a prospective cohort (*n* = 36) with capnography monitoring and matched historical controls (*n* = 36).

**Result:**

The infants had a median gestational age of 31.6 weeks. A reduction in the highest CO_2_ level on day 1 after birth was observed after the introduction of EtCO_2_ monitoring (*p* = 0.043). There was also a reduction in the magnitude of difference in CO_2_ levels on days 1 (*p* = 0.002) and 4 (*p* = 0.049) after birth. There was no significant difference in the number of blood gases.

**Conclusion:**

Continuous end-tidal capnography monitoring in ventilated infants was associated with a reduction in the degree of the magnitude of difference in CO_2_ levels and highest level of CO_2_ on the first day after birth.

## Introduction

Mechanical ventilation can be lifesaving for neonates, but long-term complications are increased in infants who have suffered abnormalities in carbon dioxide (CO_2_) levels. Disturbances in cerebral blood flow caused by the magnitude of difference in CO_2_ levels and abnormalities in CO_2_ levels can lead to intraventricular haemorrhage (IVH) and periventricular leukomalacia (PVL) and subsequent cerebral injury [1, 2]. Furthermore, outcomes of term-born neonates exposed to high CO_2_ variability during therapeutic hypothermia have been associated with adverse neurodevelopment at follow up [3]. The recent European Consensus Guidelines recommend that clinicians should avoid abnormal levels of CO_2_ by regular or continuous assessment of CO_2_ levels [4].

The current gold standard for monitoring CO_2_ levels in newborn infants is by arterial blood gas analysis [5]. This is the most accurate measurement of CO_2_ levels, however, it is not without complications [6]. Indwelling catheters in neonates can be associated with an increased risk of infection and thrombosis [7]. Frequent blood sampling can also be painful for infants when there is no indwelling arterial catheter. MRI studies have found that early exposure to pain stress in preterm infants is associated with reduced white matter and subcortical brain matter maturation [8]. Follow up of extremely preterm infants exposed to multiple heel prick tests in the neonatal period has shown that this can result in hyperalgesia and increased sensitivity to painful stimuli [9, 10]. Repeated blood sampling in neonates can lead to iatrogenic anaemia and the requirement for multiple blood transfusions, which are not without risks [11–13]. The Canadian Paediatric Society, Foetus and Newborn Committee recommend non-invasive CO_2_ monitoring for ventilated, preterm infants to minimise blood loss and the need for multiple transfusions [14].

Non-invasive end-tidal carbon dioxide monitoring by capnography is an alternative method of assessing CO_2_ levels. Capnography assesses breath by breath the exhaled carbon dioxide in real time and gives a continuous waveform together with the end-tidal CO_2_ (EtCO_2_) level [15]. Use of capnography may, therefore, reduce large magnitude of difference in CO_2_ levels. Indeed, one study found the use of continuous capnography during the resuscitation of mechanically ventilated term lambs with meconium aspiration syndrome reduced the degree of the magnitude of difference in CO_2_ levels [16]. Mainstream or sidestream capnography can be used. We recently reported on the performance of a novel microstream sidestream capnography device in ventilated newborns and found a good correlation of the results of that device with the gold standard mainstream capnograph [17].

We hypothesised that the introduction of sidestream capnography would reduce the magnitude of difference in CO_2_ levels in mechanically ventilated newborns in the first week after birth and that the number of blood samples would be reduced. We also aimed to determine whether use of sidestream capnography was associated with a reduction in severe intraventricular haemorrhage.

## Subjects and methods

The study was conducted on the neonatal intensive care unit at King’s College Hospital NHS Foundation Trust (KCH), London UK. Data were collected from a retrospective cohort of ventilated infants (historical controls) cared for in the NICU between 01/01/2017 and 01/01/2019, prior to the addition of end-tidal CO_2_ monitoring. Prospective data were collected from a cohort of ventilated infants in whom end-tidal CO_2_ monitoring was used from 01/01/2019 to 01/07/2020. Data were collected from 200 consecutively born infants admitted to the neonatal intensive care unit from 1/1/17 to 1/1/19, 36 infants from this sample were matched individually for gestational age and birth weight with the 36 infants prospectively recruited. The first infant that fulfilled the matching criteria in reverse chronological order was selected as a matched control. Approval for the study was given by the London (Camden & King’s Cross) Research Ethics Committee (REC reference: 18/LO/1602). Written informed consent was given by parents for their infants to take part in the prospective study. The retrospective data collection was registered with the clinical governance department of King’s College Hospital NHS foundation trust for retrospective use of routinely recorded data on the medical and nursing charts. No minimum number of days of invasive ventilation was required to be included into the study. Infants without full CO_2_ data were excluded as were infants that were not receiving conventional mechanical ventilation.

Infants in both cohorts were supported by volume-targeted or pressure-controlled time-cycled conventional ventilation using the SLE6000 neonatal ventilator (SLE, Croydon, UK). Infants were invasively ventilated using either volume-targeted or pressure-controlled time-cycled modes, which included assist control ventilation, synchronized intermittent mandatory ventilation or conventional mechanical ventilation. If receiving volume-targeting modes, initial targeted tidal volumes (TTV) were set at 5 ml/kg with peak inspiratory pressure maximum (PIP_max_) at 20–25 cmH_2_O, PIP_max_ was adjusted to 5 cmH_2_O above what was required to achieve the TTV according to observation of chest movement and blood gas analysis. The positive end-expiratory pressure (PEEP) was set at 5 cmH_2_O. Routine clinical practice was to adjust the ventilation settings to achieve target blood gases initially of pH 7.35–7.45 and PaCO_2_ between 4.5 and 7 kPa in the first week after birth. As per unit policy, infants were intubated with a Cole’s shouldered endotracheal tube which minimizes leak [18]. In the prospective cohort, the Microstream sidestream filterline H set sidestream capnography sample line (Phillips Medical Systems, Oridion Medical Ltd) was fitted into the ventilator circuit between the ventilator tubing and the endotracheal tube. The sample line adaptor had a deadspace of less than 0.5 mls and a weight of 3.8 g. The sample line connected to the MicroPod, an external EtCO_2_ module, containing the CO_2_ nondispersive infrared spectroscopy sensor. Gas was sampled at the proximal end of the endotracheal tube at a rate of 50 mls/minute to the MicroPod. The module performed automatic adjustment for the changes in ambient temperature. A continuous time-based capnography waveform and EtCO_2_ value was displayed on the ventilator monitor.

Arterial or capillary blood gas samples in both cohorts were taken according to the clinical need of the infant and decided by the clinicians [19]. The clinicians were not blinded to the continuous end-tidal CO_2_ monitoring which was displayed on the ventilator screen for the prospective cohort of infants. Continuous end-tidal CO_2_ monitoring was introduced as an adjunct monitoring tool and clinical staff were not instructed to change their clinical practice on the basis of end-tidal capnography alone. The results of PCO_2_ levels and the timings of blood samples were documented on the hourly nursing observation charts. The blood gas analyser used was the ABL90 FLEX PLUS analyzer (Radiometer UK Ltd.) Our outcomes included the highest and lowest PCO_2_ level and the degree of the magnitude of difference in CO_2_ levels of PCO_2_ levels (delta PCO_2_) on each day of invasive ventilation in the first week after birth. Delta PCO_2_ was calculated from blood gases values as the difference between the highest and lowest PCO_2_ values on each day.

The following data were collected from the medical and nursing notes: gestational age at birth, birth weight, gender, mode of ventilation, number of blood gas samples taken per day, the highest and lowest blood gas PCO_2_ levels per day and severe (grades 3 or 4) IVH. Data regarding the number of blood gases and levels of PCO_2_ for the prospective cohort of infants were only included if the sidestream end-tidal CO_2_ device was incorporated for the whole duration of each 24-h period of invasive ventilation so that consistent exposure to EtCO_2_ could be assessed.

### Analysis

The data were tested for normality using the Kolmogorov–Smirnov test and found not to be normally distributed. Differences between the matched pairs of infants with and without end-tidal CO_2_ monitoring, therefore, were assessed for statistical significance using the Wilcoxon paired rank sum test. Statistical analyses were undertaken using the SPSS software version 25.0 (SPSS Inc., Chicago IL).

## Results

Seventy-two infants were included in the study, 36 in the prospective group with end-tidal CO_2_ monitoring and 36 in the historical control group. Not all the infants remained ventilated for all 7 days (Table 1). The infants in the control group had a median gestational age of 31.6 (26.2–38.1) weeks and a birthweight of 1.51 (0.90–2.91) kg. The infants in the prospective cohort had a median gestational age of 31.6 (25.9–38.2) weeks and a birthweight of 1.35 (0.83–3.08) kg. There was no significant difference in the modes of ventilation used in the two cohorts (*p* = 1.0).

**Table 1:** Demographics and daily results for the full cohort of infants.

	Retrospective (*n* = 36)	Prospective (*n* = 36)	*p* value
Gestational age (weeks)	31.6 (26.2–38.1)	31.6 (25.9–38.2)	0.327
Birthweight (kg)	1.51 (0.90–2.91)	1.35 (0.83–3.08)	0.177
Male gender	19 (52.8)	18 (52.9)	1.000
Antenatal steroids (Y)	23 (63.9)	22 (69.4)	0.687
MgSO4 (Y)	13 (43.3)	14 (38.9)	1.000
Postnatal surfactant (Y)	23 (65.7)	21 (60.0)	1.000
BPD 36 weeks (Y)	14 (40.0)	15 (42.9)	1.000
Home oxygen (Y)	4 (12.1)	6 (16.7)	0.375
IVH (grades 3–4)	2 (5.6)	1 (2.9)	1.000
Survival to discharge (Y)	32 (94.1)	30 (93.8)	1.000
Day 1 (*n* = 32)			
Highest PCO_2_ kPa	7.7 (6.0–9.2)	5.8 (5.3–6.7)	0.043
Lowest PCO_2_ kPa	4.5 (3.4–5.9)	4.3 (3.6–4.8)	0.898
Delta PCO_2_ kPa	3.7 (1.8–6.2)	1.4 (1.1–3.0)	0.002
Number blood gases	3.5 (3.0–6.0)	3.0 (2.0–4.0)	0.249
Day 2 (*n* = 23)			
Highest PCO_2_ kPa	6.3 (5.4–7.3)	5.6 (5.0–6.9)	0.910
Lowest PCO_2_ kPa	4.5 (3.6–5.0)	4.3 (3.8–5.1)	0.266
Delta PCO_2_ kPa	2.0 (1.6–2.8)	1.4 (0.6–2.7)	0.197
Number blood gases	5.0 (4.0–7.0)	4.0 (2.5–6.5)	0.225
Day 3 (*n* = 20)			
Highest PCO_2_ kPa	5.7 (5.3–6.8)	6.7 (5.6–7.5)	0.014
Lowest PCO_2_ kPa	4.7 (4.1–5.3)	4.7 (4.0–5.1)	0.195
Delta PCO_2_ kPa	1.1 (0.8–2.8)	2.0 (1.0–3.0)	0.844
Number blood gases	5.0 (2.3–6.0)	4.0 (4.0–7.0)	0.293
Day 4 (*n* = 15)			
Highest PCO_2_ kPa	6.3 (5.6–6.5)	5.8 (5.1–6.7)	0.574
Lowest PCO_2_ kPa	4.6 (3.8–5.2)	4.8 (4.5–5.2)	0.102
Delta PCO_2_ kPa	1.5 (0.5–2.6)	1.1 (0.6–1.5)	0.049
Number blood gases	5.0 (4.0–6.0)	5.0 (4.0–5.0)	0.449
Day 5 (*n* = 13)			
Highest PCO_2_ kPa	6.7 (5.9–7.6)	5.9 (5.2–6.8)	1.000
Lowest PCO_2_ kPa	5.0 (4.4–5.4)	5.9 (4.1–5.6)	0.484
Delta PCO_2_ kPa	1.7 (0.9–2.7)	1.0 (0.7–3.2)	1.000
Number blood gases	5.0 (2.5–7.0)	5.0 (3.0–5.8)	0.938
Day 6 (*n* = 11)			
Highest PCO_2_ kPa	6.5 (5.8–7.3)	6.2 (5.5–7.4)	0.250
Lowest PCO_2_ kPa	5.3 (4.8–6.0)	5.5 (4.7–6.0)	1.000
Delta PCO_2_ kPa	1.0 (0.5–2.6)	1.0 (0.5–2.0)	0.875
Number blood gases	4.0 (3.0–6.0)	4.5 (1.8–6.0)	0.250
Day 7 (*n* = 7)			
Highest PCO_2_ kPa	7.6 (7.3–8.0)	7.0 (6.4–8.2)	0.875
Lowest PCO_2_ kPa	5.8 (5.5–6.0)	6.0 (4.7–6.5)	0.875
Delta PCO_2_ kPa	1.7 (1.2–2.0)	1.1 (0.6–1.7)	0.875
Number blood gases	5.0 (3.0–6.0)	5.0 (3.0–7.3)	0.125

1 kPa = 7.5 mmHg.

Data are presented as median (IQR) or *n* (%).

There was a significant reduction in the highest PCO_2_ measured on arterial or capillary blood gases in the prospective group compared to the historical control group on day 1 after birth [5.8 (5.3–6.7) kPa versus 7.7 (6.0–9.2) kPa; *p* = 0.043]. There was also a significant reduction in the delta PCO_2_ levels on day 1 after birth [1.4 (1.1–3.0) kPa versus 3.7 (1.8–6.2) kPa; *p* = 0.002] and on day 4 [1.5 (0.5–2.6) kPa versus 1.1 (0.6–1.5) kPa; *p* = 0.049].

No significant difference was observed in the number of blood gases between those with and those without EtCO_2_ monitoring (Table 1). There was no reduction in severe (grades 3 or 4) intraventricular haemorrhage within the prospective cohort compared to the retrospective controls [2 (10.5%) versus 2 (10.5%); *p* = 1.0] or in the most prematurely born infants and at highest risk of IVH (Table 2).

**Table 2:** Demographics and daily results for the subgroup of very premature infants. Data are presented as median (IQR) or *n* (%).

<32 weeks (*n* = 38)	Retrospective	Prospective	*p* value
Gestational age (weeks)	26.3 (25.3–30.0)	26.1 (25.0–30.0)	
Birthweight (kg)	0.93 (0.72–1.29)	0.88 (0.63–1.06)	
IVH (Gr3/4)	2 (10.5)	2 (10.5)	1.000
Day 1			
Highest PCO_2_ kPa	7.9 (4.6–8.8)	6.1 (5.3–6.9)	0.240
Lowest PCO_2_ kPa	4.7 (3.8–6.2)	4.6 (4.0–5.2)	0.938
Delta PCO_2_ kPa	3.1 (1.3–4.8)	1.4 (1.2–2.9)	0.020
Number blood gases	3.0 (3.0–6.0)	3.0 (2.0–3.3)	0.438
Day 2			
Highest PCO_2_ kPa	6.7 (5.3–7.7)	5.6 (5.1–7.1)	0.375
Lowest PCO_2_ kPa	5.0 (3.6–5.3)	4.6 (3.3–5.3)	0.625
Delta PCO_2_ kPa	2.2 (3.3–6.8)	1.2 (0.8–3.0)	0.875
Number blood gases	5.0 (3.3–6.8)	4.5 (3.3–6.0)	0.625
Day 3			
Lowest PCO_2_ kPa	6.0 (5.6–7.5)	7.0 (5.7–8.0)	0.125
Delta PCO_2_ kPa	5.2 (4.5–5.4)	5.0 (4.3–5.7)	0.875
Highest PCO_2_ kPa	1.0 (0.8–2.9)	2.3 (0.9–3.8)	0.375
Number blood gases	6.0 (3.5–6.3)	4.0 (4.0–7.0)	0.500
Day 4			
Highest PCO_2_ kPa	6.4 (5.9–6.8)	6.2 (5.2–6.7)	0.750
Lowest PCO_2_ kPa	4.6 (3.8–5.1)	5.0 (4.5–5.7)	0.094
Delta PCO_2_ kPa	1.7 (0.9–2.8)	0.9 (0.3–1.4)	0.156
Number blood gases	5.0 (3.5–6.0)	5.0 (4.0–5.0)	0.437
Day 5			
Highest PCO_2_ kPa	7.2 (6.4–7.8)	6.3 (5.3–8.3)	0.750
Lowest PCO_2_ kPa	5.0 (4.9–5.4)	5.1 (4.5–5.6)	1.000
Delta PCO_2_ kPa	2.3 (1.2–2.8)	0.9 (0.5–3.3)	0.750
Number blood gases	6.0 (5.0–7.0)	4.0 (3.0–5.0)	0.500
Day 6			
Highest PCO_2_ kPa	7.0 (6.5–8.1)	7.7 (6.1–7.8)	0.500
Lowest PCO_2_ kPa	5.6 (4.9–6.6)	5.6 (5.4–6.1)	1.000
Delta PCO_2_ kPa	2.1 (0.7–2.7)	1.7 (0.7–2.0)	0.500
Number blood gases	5.0 (3.0–6.0)	6.0 (4.0–8.0)	1.000
Day 7			
Highest PCO_2_ kPa	7.7 (7.4–8.0)	7.5 (5.9–8.3)	0.750
Lowest PCO_2_ kPa	5.9 (5.5–6.3)	6.1 (4.6–6.9)	1.000
Delta PCO_2_ kPa	1.7 (1.3–2.2)	1.2 (0.6–2.3)	1.000
Number blood gases	5.0 (3.8–6.5)	7.0 (3.5–8.0)	0.250

1 kPa = 7.5 mmHg

## Discussion

Continuous, real-time sidestream capnography was associated with a reduction in the degree of the magnitude of difference in CO_2_ levels and highest level of carbon dioxide on the first day after birth in mechanically ventilated infants.

We have reported that abnormal levels of carbon dioxide, including large magnitude of difference in CO_2_ levels during resuscitation contribute to IVH development [20]. A previous retrospective study reported the maximum PCO_2_ during the first 72 hours after birth was a dose-dependent predictor of severe IVH development [21]. Continuous monitoring of EtCO_2_ may, therefore, have the potential to reduce the complications associated with abnormal PCO_2_ levels. The low rates of IVH and the lack of significant difference in IVH rates between the two groups in our study may reflect that despite tighter control of carbon dioxide levels in the prospective cohort, the median PCO_2_ values in both cohorts were still maintained within the recommended range for preterm infants on invasive ventilation [22]. We appreciate that our sample size, particularly in the sub-analysis, did not allow us to robustly assess whether the monitoring had an impact on the incidence of IVH, but this was not our primary objective.

A reduction in the magnitude of difference in CO_2_ levels of PCO_2_ levels on day 1 after birth in the whole prospective cohort and on day 4 in the infants is in agreement with a previous study using distal EtCO_2_ monitoring [23]. That study, however, had a primary outcome of the time spent within a predefined range of PCO_2_ rather than the magnitude of difference in CO_2_ levels of PCO_2_ values. Distal EtCO_2_ monitoring, however, is not widely used in the neonatal population.

In a retrospective study in a paediatric intensive care unit, a reduction in the total number of blood gas analyses was found, following the introduction of continuous sidestream capnography [24]. The change in clinician behaviour, however, was over a 3-year period [24]. The introduction of transcutaneous CO_2_ monitoring in ventilated infants, despite only showing a moderate agreement with PCO_2_ values and no decrease in rates of IVH, was associated with a reduction in the frequency of blood gas sampling [25], with the reduction being more pronounced in infants receiving high frequency oscillatory ventilation (HFOV). In this study, there was no reduction in the frequency of blood gas sampling following the introduction of capnography monitoring. It may be noted that our study period was too short for an effect on behaviour to have occurred or the display of continuous EtCO_2_ levels may have prompted clinicians to undertake confirmatory blood gases if extremes were noted and hence keeping infants within a narrower range of PCO_2_.

There has been some scepticism amongst clinicians regarding the use of capnography in neonates [26] due to their small tidal volumes and rapid respiratory rates [27]. This may, therefore, affect how they interpret its accuracy and hence their willingness to reduce the number of blood gases taken to monitor PCO_2_. We have recently described end -tidal CO_2_ is strongly correlated with PCO_2_ and the two methods only diverge, as expected, with increasing severity of the underlying lung pathology [17].

Our study has strengths and some limitations. The non-invasive microstream sidestream capnograph device used in the present study has been previously validated against mainstream capnography [17]. We included preterm and term infants who required ventilation for a wide range of pathologies and hence results from this study can be generalisable to other medical and surgical tertiary neonatal intensive care units. We feel our sample size was appropriate, as with a standard deviation for carbon dioxide of 14.8 mmHg (1.97 kpa) in preterm infants [1] our sample of 36 infants in each arm (72 infants in total) could detect a difference in carbon dioxide of 11.3 mmHg (1.51 kPa) with 90% power at 5% significance level. This difference is smaller than the clinically significant difference in carbon dioxide of 13.0 mmHg (1.73 kPa) reported between preterm infants with or without IVH [1]. This was not a randomised trial, but the two time periods studied, pre and post introduction of EtCO_2_ monitoring, were consecutive and no other major changes in clinical practice occurred during the study period. A possible limitation of our study was that we routinely used Coles shouldered tubes which have been shown to have minimal or no leak [18] and hence may enable more accurate EtCO_2_ measurements. Such tubes are not in widespread practice and it would be interesting to assess if the type of endotracheal tube does influence the accuracy of EtCO_2_ monitoring

In conclusion, we have demonstrated that the presence of continuous end-tidal capnography monitoring in ventilated newborns was associated with a reduction in the highest level and the magnitude of difference in CO_2_ levels within the first day after birth.

## Data Availability

Data available upon request.

## References

[1] Fabres J, Carlo WA, Philips V, Howard G, Ambalavanan N (2007). Both extremes of arterial carbon dioxide pressure and the magnitude of fluctuations in arterial carbon dioxide pressure are associated with severe intraventricular haemorrhage in preterm infants. Pediatrics.

[2] Okumura A, Hayakawa F, Kato T, Itomi K, Maruyama K, Ishihara N (2001). Hypocarbia in preterm infants with periventricular leukomalacia: the relation between hypocarbia and mechanical ventilation. Pediatrics.

[3] Hansen G, Al Shafouri N, Narvey M, Vallance JK, Srinivasan G (2016). High blood carbon dioxide variability and adverse outcomes in neonatal hypoxic ischemic encephalopathy. J Matern Fetal Neonatal Med.

[4] Sweet DG, Carnielli V, Greisen G, Hallman M, Ozek E, Te Pas A (2019). European consensus guidelines on the management of respiratory distress syndrome - 2019 Update. Neonatology.

[5] Singh BS, Gilbert U, Singh S, Govindaswami B (2013). Sidestream microstream end tidal carbon dioxide measurements and blood gas correlations in neonatal intensive care unit. Pediatr Pulmonol.

[6] Trevisanuto D, Giuliotto S, Cavallin F, Doglioni N, Toniazzo S, Zanardo V (2012). End-tidal carbon dioxide monitoring in very low birth weight infants: correlation and agreement with arterial carbon dioxide. Pediatr Pulmonol.

[7] Levit OL, Shabanova V, Bizzarro MJ (2020). Umbilical catheter-associated complications in a level IV neonatal intensive care unit. J Perinatol.

[8] Brummelte S, Grunau RE, Chau V, Poskitt KJ, Brant R, Vinall J (2012). Procedural pain and brain development in premature newborns. Ann Neurol.

[9] Walker SM, Franck LS, Fitzgerald M, Myles J, Stocks J, Marlow N (2009). Long-term impact of neonatal intensive care and surgery on somatosensory perception in children born extremely preterm. Pain.

[10] Grunau RE, Oberlander TF, Whitfield MF, Fitzgerald C, Morison SJ, Saul JP (2001). Pain reactivity in former extremely low birth weight infants at corrected age 8 months compared with term born controls. Infant Behav Dev.

[11] Jakacka N, Snarski E, Mekuria S (2016). Prevention of iatrogenic anemia in critical and neonatal care. Adv Clin Exp Med.

[12] Papa F, Rongioletti M, Ventura MD, Di Turi F, Cortesi M, Pasqualetti P (2011). Blood cell counting in neonates: a comparison between a low volume micromethod and the standard laboratory method. Blood Transfus.

[13] Strauss RG (1991). Transfusion therapy in neonates. Am J Dis Child.

[14] Lemyre B, Sample M, Lacaze-Masmonteil T, Canadian Paediatric Society F, Newborn C. (2015). Minimizing blood loss and the need for transfusions in very premature infants. Paediatr Child Health.

[15] Gravenstein JS Capnography. 2nd ed. Cambridge. Cambridge University Press: New York 2011.

[16] Chandrasekharan PK, Rawat M, Nair J, Gugino SF, Koenigsknecht C, Swartz DD (2016). Continuous end-tidal carbon dioxide monitoring during resuscitation of asphyxiated term lambs. Neonatology.

[17] Williams E, Dassios T, Greenough A (2020). Assessment of sidestream end-tidal capnography in ventilated infants on the neonatal unit. Pediatr Pulmonol.

[18] Hird M, Greenough A, Gamsu H (1990). Gas trapping during high frequency positive pressure ventilation using conventional ventilators. Early Hum Dev.

[19] McLain BI, Evans J, Dear PR (1988). Comparison of capillary and arterial blood gas measurements in neonates. Arch Dis Child.

[20] Tamura K, Williams EE, Dassios T, Pahuja A, Hunt KA, Murthy V (2020). End-tidal carbon dioxide levels during resuscitation and carbon dioxide levels in the immediate neonatal period and intraventricular haemorrhage. Eur J Pediatr.

[21] Kaiser JR, Gauss CH, Pont MM, Williams DK (2006). Hypercapnia during the first 3 days of life is associated with severe intraventricular hemorrhage in very low birth weight infants. J Perinatol.

[22] Specialist neonatal respiratory care for babies born preterm. National Institute for Health and Care Excellence: clinical guidelines. London 2019. 31194313

[23] Kugelman A, Golan A, Riskin A, Shoris I, Ronen M, Qumqam N (2016). Impact of continuous capnography in ventilated neonates: a randomized, multicenter study. J Pediatr.

[24] Rowan CM, Speicher RH, Hedlund T, Ahmed SS, Swigonski NL (2015). Implementation of continuous capnography is associated with a decreased utilization of blood gases. J Clin Med Res.

[25] Mukhopadhyay S, Maurer R, Puopolo KM (2016). Neonatal transcutaneous carbon dioxide monitoring-effect on clinical management and outcomes. Respir Care.

[26] Cook TM, Kelly FE, Foy K, Mew E, Bower J, Marden B (2019). The PIC-NIC survey: capnography and neonatal intensive care - a reply. Anaesthesia.

[27] Schmalisch G (2016). Current methodological and technical limitations of time and volumetric capnography in newborns. Biomed Eng Online.

