# Correction: End-tidal capnography monitoring in infants ventilated on the neonatal intensive care unit

**DOI:** 10.1038/s41372-021-01218-z

**Published:** 2021-10-07

**Authors:** Emma Williams, Theodore Dassios, Niamh O’Reilly, Alison Walsh, Anne Greenough

**Affiliations:** 1 Department of Women and Children’s Health, School of Life Course Sciences, Faculty of Life Sciences and Medicine, King’s College London, London, UK; 2 The Asthma UK Centre for Allergic Mechanisms in Asthma, King’s College London, London, UK; 3 Neonatal Intensive Care Unit, King’s College Hospital NHS Foundation Trust, London, UK; 4 NIHR Biomedical Research Centre based at Guy’s and St Thomas’ NHS Foundation Trust, King’s College London, London, UK

**Keywords:** Risk factors, Outcomes research

Correction to: *Journal of Perinatology* 10.1038/s41372-021-00978-y, published online 01 March 2021

The article End-tidal capnography monitoring in infants ventilated on the neonatal intensive care unit, written by EW, TD, NOR, AW, and AG was originally published Online First without Open Access. After publication in volume 41, issue 7, page 1718–1724 the author decided to opt for Open Choice and to make the article an Open Access publication. Therefore, the copyright of the article has been changed to © The Author(s) 2021 and the article is forthwith distributed under the terms of the Creative Commons Attribution 4.0 International License, which permits use, sharing, adaptation, distribution, and reproduction in any medium or format, as long as you give appropriate credit to the original author(s) and the source, provide a link to the Creative Commons licence, and indicate if changes were made.

The images or other third party material in this article are included in the article’s Creative Commons licence, unless indicated otherwise in a credit line to the material. If material is not included in the article’s Creative Commons licence and your intended use is not permitted by statutory regulation or exceeds the permitted use, you will need to obtain permission directly from the copyright holder.

